# Are hedonic hunger and health-related quality of life associated with obesity in adolescents?

**DOI:** 10.3389/fnut.2025.1557765

**Published:** 2025-03-26

**Authors:** Bilge Meral Koc, Emre Batuhan Kenger, Ezgi Arslan Yuksel, Tugce Ozlu Karahan

**Affiliations:** ^1^Department of Nutrition and Dietetics, Faculty of Health Sciences, Izmir Democracy University, Izmir, Türkiye; ^2^Department of Nutrition and Dietetics, Faculty of Health Sciences, Istanbul Bilgi University, Istanbul, Türkiye; ^3^Department of Nutrition and Dietetics, Faculty of Health Sciences, Halic University, Istanbul, Türkiye

**Keywords:** adolescent, fast food, hedonic hunger, health-related quality of life, sugary drinks

## Abstract

The purpose of the study is to find out the relationship between obesity, hedonic hunger and health-related quality of life (HRQoL). The questionnaire containing items on the sociodemographic characteristics, anthropometric measurements and nutritional habits of individuals (*n* = 902), the Children’s Power of Food Scale and the KIDSCREEN-10 were used to determine the hedonic hunger and the HRQoL score. Hedonic hunger and HRQoL were evaluated according to the categories, frequency of consumption of sugary drinks and fast food. The females were found to have hedonic hunger more than the males (*p* < 0.05). Body mass index z-score categories of the adolescents showed that the obese eat more hedonically compared to the underweight and the normal-weight (*p* < 0.05), and this difference was not observed in the HRQoL scores (*p* > 0.05). There was a negative correlation between the hedonic hunger status and the HRQoL scores (*R* = −0.342, *p* = 0.000). The consumption of sugary drinks and fast food was associated with hedonic hunger and HRQoL scores in certain groups. Hedonic hunger is associated with obesity; hedonic hunger and quality of life are important factors associated with unhealthy food and drink intake.

## Introduction

Childhood obesity is a disorder of energy balance that develops due to multifactorial causes. Obesity may develop as a result of disturbance in the energy balance due to homeostatic mechanisms, as well as emotional relaxation as a result of emotional hunger or excessive energy intake due to the rewarding feature of food (1). Examples of reward-based eating include food addiction, attentional bias on food, and hedonic hunger. Hedonic hunger is defined as having “frequent thoughts, feelings, and urges about food in the absence of short- or long-term energy deficit” (2, 3). Eating in the absence of physiological hunger is shown as a behavioral disorder. Eating for pleasure or eating without hunger, that is, hedonic hunger, has an important place in the etiology of obesity (4). This hedonistic aspect of nutrition is regulated by the corticolimbic neural system and the psychological effects of food intake such as motivational and emotional ones. Available diversity of food also contributes to hedonic hunger. Catching the sight, smell and feeling of foods are reported as food cues of hedonic hunger. It was shown that the triggered responses to these food cues are higher in slightly overweight and obese individuals than in healthy individuals (5). A study conducted with individuals with different body mass index (BMI) reported that hedonic hunger is associated with eating behavior, and behavior disorder related to hedonic hunger is more common in obese individuals (6). Various studies have been conducted on hedonic hunger and food intake in adolescents. Cross-sectional data have shown a positive relationship between hedonic hunger and unhealthy food consumption (2, 7). However, the relationships explored in this analysis did not specifically examine the connection between quality of life and hedonic hunger.

Health-related quality of life (HRQoL) is a concept that incorporates physical, psychological and social health (8). A comprehensive study conducted in 12 countries found that children aged 9–11 who prefer healthy food have higher HRQoL scores. A recent systematic review study (*n* = 17 studies) stated that higher diet quality is associated with higher HRQoL scores in children and adolescents (8). In addition, childhood obesity was reported to increase the risk of psychological and physical health problems in children and to be associated with lower HRQoL scores (9).

Although there are a few studies on hedonic hunger and health (10, 11), there is no study in the literature examining the effects of hedonic hunger on HRQoL in children and adolescents. Addressing this gap, the present study aims to determine the relationship between BMI, which is an important criterion in the evaluation/follow-up of childhood and adolescent obesity, and hedonic hunger and HRQoL. Another aim of this study is to find out whether there is a difference between overweight and normal-weight children and adolescents in terms of these factors. Thus, the relationship between obesity and hedonic hunger will be revealed.

## Material and method

The sample of this cross-sectional study consisted of 902 adolescents aged 10–17 living in Istanbul, Turkey. The power of the study was calculated using the G*Power (G*Power 3.1.9.2, Düsseldorf, Germany) software (12). The sample size was calculated in a way that type 1 error rate was *α* = 0.05 and type 2 error rate was *β* = 0.20 and the power of the test was 1- *β* = 0.80 (3). Those with diseases that might affect their mental health such as autism spectrum disorder, those who could not speak or communicate, those who were on prescription drugs within the scope of obesity treatment, those who had undergone or were planned to undergo bariatric surgeries were not included in the study in order not to affect the results.

Containing items on the sociodemographic characteristics, anthropometric measurements and nutritional habits of participants, the questionnaire was filled in online. The Children’s Power of Food Scale, which was developed for children and adolescents to determine their hedonic hunger status, and the KIDSCREEN-10, which was also developed for children and adolescents to determine their HRQoL scores, were applied within the same questionnaire. To ensure data was collected after meals, participants were instructed to complete the surveys after eating, and an item was added to the survey for participants to check and confirm compliance with this instruction.

### Hedonic hunger

The Children’s Power of Food Scale was developed by Lowe et al. (13), and the validity and reliability study of the Turkish version was performed by Sahin-Bodur et al. (14). The items were designed with a five-point Likert scale (1, strongly disagree, 5, strongly agree). Each item was scored out of five points, so the mean score were obtained by dividing the total score by the number of items. As the mean score obtained on the Children’s Power of Food Scale increases, the hedonic hunger level of participants increases. Higher scores indicate that individuals are more sensitive to the food environment and are psychologically controlled by food (14).

### Health-related quality of life (HRQoL)

A scale development project was carried out with the initiative of 13 European countries to address the need for a scale that can enable comparison between cultures and reflect the characteristics of the culture in which it was developed, despite many health quality of life scales developed for children in general and specific to the disease (15). In this project, a general-purpose health quality of life scale called KIDSCREEN-10 was developed (16). The validity and reliability of the Turkish version of KIDSCREEN-10, which was developed for children and adolescents aged 8–18 years, was conducted by Baydur et al. (17). The KIDSCREEN-10 scale is evaluated out of 50 points, and as the score increases, HRQoL of children increases, too (17).

### Data analysis

The data were analyzed using SPSS 21.0, with statistical significance set at *p* < 0.05. Descriptive statistics included count, percentage, mean, and standard deviation values. The chi-square test was used for categorical variables, while the Kolmogorov–Smirnov test assessed normality. Independent samples t-test compared hedonic hunger and HRQoL scores by gender, and one-way ANOVA tested differences across BMI z-score categories and food consumption groups. Pearson correlation analyzed relationships between hedonic hunger and HRQoL. Multiple regression analyses identified predictors of hedonic hunger and HRQoL, reporting unstandardized coefficients (B), standard errors (SE), *t*-values, *p*-values, and 95% confidence intervals (CI). Post-hoc comparisons were conducted using the Least Significant Difference (LSD) test instead of Tukey HSD, as LSD is more appropriate for detecting differences in groups with small sample sizes and provides greater statistical power. Effect sizes were calculated using Cohen’s d for continuous variables, Cramer’s V for categorical variables, and Eta-squared (η^2^) for ANOVA results.

## Results

A total of 902 adolescents with a mean age of 14.59 ± 2.01 years participated in our study. The mean BMI was 22.26 ± 11.82 kg/m^2^, and no significant difference was observed between genders (*p* = 0.624, *d* = −0.032), indicating a negligible effect size. However, the BMI z-scores of female participants were significantly lower than those of males (*p* < 0.001, *d* = −0.461), suggesting a moderate effect size. When examining hedonic hunger and quality of life, male participants experienced significantly less hedonic hunger than females (*p* < 0.001, *d* = 0.269, small effect), while their quality of life was higher (*p* < 0.001, *d* = 0.299, small-to-moderate effect). The general characteristics of the participants according to gender are summarized in Table 1.

**Table 1:** General characteristics of the participants by gender.

	Female (*n* = 559)	Male (*n* = 343)	Total (*n* = 902)	*P*	Effect size
Age	14.73 ± 1.95	14.36 ± 2.05	14.59 ± 2.01	**0.006**	**0.188**
Weight	55.01 ± 11.93	65.74 ± 18.69	59.01 ± 15.75	**<0.001**	**−0.723**
Height	160.73 ± 12.80	169.62 ± 13.20	164.11 ± 13.65	**<0.001**	**−0.746**
BMI	22.12 ± 14.50	22.51 ± 5.02	22.26 ± 11.82	0.624	−0.032
BMI-z score	0.10 ± 1.19	0.68 ± 1.35	0.32 ± 1.29	**<0.001**	**−0.461**
Hedonic hunger score	3.25 ± 0.83	3.04 ± 0.82	3.17 ± 0.83	**<0.001**	**0.269**
HRQoL	32.35 ± 7.06	34.43 ± 6.78	33.14 ± 7.02	**<0.001**	**0.299**
BMI-z score categories				**<0.001**	**0.214**
Malnutrition	21 (3.8)	5 (1.5)	26 (2.9)		
Underweight	72 (12.9)	30 (8.7)	102 (11.3)		
Normal weight	342 (61.2)	167 (48.7)	509 (56.4)		
Slightly overweight	87 (15.6)	87 (25.4)	174 (19.3)		
Obesity	37 (6.6)	54 (15.7)	91 (10.1)		
Number of main meals				**<0.001**	**0.157**
1	20 (3.6)	14 (4.1)	34 (3.8)		
2	235 (42.0)	91 (26.5)	326 (36.1)		
3	304 (54.4)	238 (69.4)	542 (60.1)		
Number of snacks				**0.004**	**0.121**
0	28 (5.0)	34 (9.9)	62 (6.9)		
1	163 (29.2)	109 (31.8)	272 (30.2)		
2	255 (45.6)	123 (35.9)	378 (41.9)		
3	113 (20.2)	77 (22.4)	190 (21.1)		
Frequency of consumption of sugary drinks (daily)				**<0.001**	**0.139**
No consumption	116 (20.8)	64 (18.7)	180 (20.0)		
Less than 2 glasses	269 (48.1)	126 (36.7)	395 (43.8)		
2 and more glasses	174 (31.1)	153 (44.6)	327 (36.2)		
Frequency of consumption of fast food (weekly)				0.106	0.071
No consumption	50 (8.9)	44 (12.8)	94 (10.4)		
Less than 2 times	325 (58.1)	180 (52.5)	505 (56.0)		
2 and more times	184 (32.9)	119 (34.7)	303 (33.7)		

Continuous variables are mentioned as mean ± standard deviation, and categorical variables as counts and percentages. Significant *p* values are indicated in bold.

**Figure 1:**
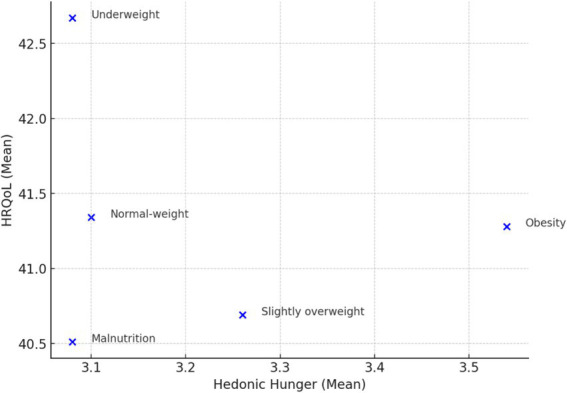
Relationship between hedonic hunger and HRQoL across BMI categories.

We examined adolescents’ hedonic hunger and quality of life according to BMI-z score categories. The results indicated a significant difference in hedonic hunger among BMI categories (*F* = 6.405, *p* < 0.001, η^2^ = 0.028), suggesting a small-to-moderate effect size. Post-hoc analyses revealed that obese adolescents (3.54 ± 0.88) experienced significantly higher hedonic hunger compared to underweight (3.08 ± 0.74, *p* < 0.001, *d* = −0.530) and normal-weight adolescents (3.10 ± 0.82, *p* < 0.001, *d* = −0.565), both with moderate effect sizes. Additionally, slightly overweight adolescents (3.27 ± 0.81) had significantly higher hedonic hunger than underweight adolescents (*p* = 0.016, *d* = −0.196), though the effect size was small. However, no significant differences were observed between malnourished, underweight, and normal-weight adolescents (*p* > 0.05). Regarding health-related quality of life (HRQoL), no statistically significant differences were found across BMI categories (*p* = 0.211, η^2^ = 0.006), indicating a negligible effect size (Table 2; Figure 1).

**Table 2:** Relationship between hedonic hunger and health-related quality of life according to BMI.

	BMI-z score categories	Mean ± SD	*F*	*P*	η^2^	Post-hoc comparisons	Cohen’s d
Hedonic hunger	Malnutrition	3.08 ± 1.00	6.522	**<0.001**	**0.028**	Obesity > Underweight (*p* < 0.001)	−0.530
	Underweight	3.08 ± 0.74				Obesity > Normal weight (*p* < 0.001)	−0.565
	Normal weight	3.10 ± 0.82				Slightly overweight > Underweight (*p* < 0.001)	−0.196
	Slightly overweight	3.26 ± 0.81					
	Obesity	3.54 ± 0.88					
HRQoL	Malnutrition	32.04 ± 8.18	1.466	0.211	0.006	No significant differences	
	Underweight	34.29 ± 6.86					
	Normal weight	33.29 ± 6.84					
	Slightly overweight	32.60 ± 7.16					
	Obesity	32.33 ± 7.49					

HRQoL: Health-related quality of life. Significant *p* values are indicated in bold.

The adolescents were evaluated based on their daily sugary drink consumption and its effect on hedonic hunger and HRQoL across BMI-z score categories. Among underweight participants, those who did not consume sugary drinks (37.65 ± 4.68) had a significantly higher HRQoL than those consuming two or more glasses per day (32.41 ± 7.25, *p* = 0.006, η^2^ = 0.075), with a large effect size (Cohen’s *d* = 0.81). In the normal-weight category, a significant difference was observed in hedonic hunger (*p* = 0.002, η^2^ = 0.022, small effect size). Adolescents who consumed two or more glasses daily (3.27 ± 0.81) experienced greater hedonic hunger compared to both those with no consumption (2.94 ± 0.85, *p* = 0.002, *d* = 0.40, small-to-moderate effect) and those consuming less than two glasses per day (3.05 ± 0.81, *p* = 0.014, *d* = 0.27, small effect). For HRQoL in the normal-weight category, adolescents who did not consume sugary drinks (34.56 ± 6.56) had significantly higher HRQoL than those consuming two or more glasses daily (32.38 ± 6.74, *p* = 0.011, *d* = 0.33, small-to-moderate effect). In slightly overweight and obese adolescents, no significant differences were found in hedonic hunger or HRQoL across sugary drink consumption groups (*p* > 0.05), indicating a negligible effect of sugary drink intake in these categories. These findings suggest that higher sugary drink consumption is associated with greater hedonic hunger and lower HRQoL, particularly in underweight and normal-weight adolescents. The large effect size in underweight adolescents highlights a notable impact of sugary drink intake on quality of life, while small-to-moderate effects in normal-weight adolescents indicate a meaningful but less pronounced association (Table 3).

**Table 3:** Hedonic hunger and health-related quality of life according to consumption of sugary drinks.

BMI-z score categories		Frequency of consumption of sugary drinks (daily)			*F*	*p*	η^2^	Post-hoc comparisons	Cohen’s d
		No consumption	Less than 2 glasses	2 and more glasses					
Malnutrition	n	6	9	11					
	Hedonic hunger	2.76 ± 0.96	3.26 ± 1.06	3.10 ± 1.02	0.435	0.652	0.037		
	HRQoL	28.18 ± 7.05	33.11 ± 7.66	33.27 ± 9.14	4.025	0.434	0.070		
Underweight	n	20	45	37					
	Hedonic hunger	3.12 ± 0.64	2.92 ± 0.75	3.24 ± 0.75	1.945	0.148	0.038		
	HRQoL	37.65 ± 4.68	34.36 ± 6.88	32.41 ± 7.25	4.025	**0.021**	**0.075**	No consumption >2 and more glasses (p = 0.006)	0.081
Normal weight	n	106	234	169					
	Hedonic hunger	2.94 ± 0.85	3.05 ± 0.81	3.27 ± 0.81	6.103	**0.002**	**0.022**	2 and more glasses > No consumption (p = 0.002)	0.33
								2 and more glasses > Less than 2 glasses (p = 0.014)	0.27
	HRQoL	34.56 ± 6.56	33.36 ± 6.97	32.38 ± 6.74	3.370	**0.035**	**0.013**	No consumption >2 and more glasses (p = 0.011)	0.40
Slightly overweight	n	33	76	65					
	Hedonic hunger	3.01 ± 1.00	3.29 ± 0.73	3.36 ± 0.77	2.179	0.116	0.026		
	HRQoL	34.91 ± 7.95	32.44 ± 6.86	31.64 ± 6.94	2.369	0.097	0.027		
Obesity	n	15	31	45					
	Hedonic hunger	3.42 ± 1.06	3.38 ± 0.65	3.70 ± 0.94	1.069	0.256	0.032		
	HRQoL	31.93 ± 8.72	33.38 ± 6.44	31.71 ± 7.85	0.480	0.621	0.011		

HRQoL: Health-related quality of life. Significant *p* values are indicated in bold.

The relationship between fast food consumption, hedonic hunger, and health-related quality of life (HRQoL) was evaluated across BMI-z score categories. Among slightly overweight adolescents, those who did not consume fast food (2.78 ± 0.84) experienced significantly lower hedonic hunger than those consuming it less than 2 times per week (3.23 ± 0.78, *p* = 0.030, *d* = 0.57, moderate effect) and those consuming it 2 or more times per week (3.50 ± 0.76, *p* < 0.001, *d* = 0.92, large effect). Similarly, those consuming fast food less than 2 times per week had lower hedonic hunger than those consuming it 2 or more times per week (*p* = 0.016, *d* = 0.35, small-to-moderate effect).

A significant difference was also observed in normal-weight adolescents (*p* < 0.001, η^2^ = 0.072). Those consuming fast food 2 or more times per week (3.38 ± 0.84) had significantly higher hedonic hunger compared to those consuming it less than 2 times per week (3.02 ± 0.76, *p* < 0.001, *d* = 0.46, moderate effect) and those who did not consume fast food (2.70 ± 0.89, *p* < 0.001, *d* = 0.80, large effect). Additionally, adolescents consuming fast food less than 2 times per week had higher hedonic hunger than those with no consumption (*p* = 0.006, *d* = 0.41, small-to-moderate effect).

Regarding HRQoL, a significant difference was found in the normal-weight category (*p* < 0.001, η^2^ = 0.034). Adolescents who did not consume fast food (35.89 ± 7.18) had significantly higher HRQoL than those consuming it less than 2 times per week (33.66 ± 6.53, *p* = 0.026, d = 0.34, small-to-moderate effect) and those consuming it 2 or more times per week (31.66 ± 6.97, *p* < 0.001, *d* = 0.60, moderate-to-large effect). Additionally, those consuming fast food less than 2 times per week had higher HRQoL than those consuming it 2 or more times per week (*p* = 0.003, *d* = 0.30, small effect).

For obese adolescents, a significant difference in HRQoL was observed (*p* = 0.011, η^2^ = 0.097). Those who did not consume fast food (35.75 ± 7.21) had significantly higher HRQoL than those consuming it 2 or more times per week (30.20 ± 6.62, *p* = 0.008, d = 0.83, large effect). Similarly, those consuming fast food less than 2 times per week had higher HRQoL than those consuming it 2 or more times per week (*p* = 0.046, d = 0.62, moderate-to-large effect). These findings indicate that frequent fast-food consumption is associated with decreased HRQoL, particularly in normal-weight and adolescents with obesity. And also frequent fast-food consumption is associated with increased hedonic hunger in normal weight and slightly overweight, The large effect sizes in slightly overweight and obese groups highlight the strong impact of fast-food consumption on both psychological and quality of life outcomes, emphasizing the need for dietary interventions among adolescents (Table 4).

**Table 4:** Hedonic hunger and health-related quality of life by fast food consumption.

BMI z-score categories		Frequency of consumption of fast food (weekly)			*F*	*p*	η^2^	Post-hoc comparisons	Cohen’s d
		No consumption	Less than 2 times	2 or more times					
Malnutrition	n	5	10	11					
	Hedonic hunger	2.50 ± 0.91	2.88 ± 0.95	3.53 ± 0.95	2.391	0.114	0.172		
	HRQoL	32.80 ± 6.10	34.40 ± 9.51	29.55 ± 7.62	0.946	0.403	0.076		
Underweight	n	5	66	31					
	Hedonic hunger	2.72 ± 0.25	3.04 ± 0.75	3.20 ± 0.75	1.123	0.330	0.022		
	HRQoL	38.00 ± 10.89	34.42 ± 5.96	33.42 ± 7.91	0.993	0.374	0.020		
Normal-weight	n	54	300	155					
	Hedonic hunger	2.70 ± 0.89	3.02 ± 0.76	3.38 ± 0.84	17.495	**<0.001**	**0.072**	2 or more times > No consumption (*p* < 0.001)	0.080
								2 or more times > Less than 2 times (*p* < 0.001)	0.046
								Less than 2 times > No consumption (*p* = 0.006)	0.041
	HRQoL	35.89 ± 7.18	33.66 ± 6.53	31.66 ± 6.97	9.024	**<0.001**	**0.034**	No consumption > Less than 2 times (*p* = 0.026)	0.34
								No consumption >2 or more times (*p* < 0.001)	0.60
								Less than 2 times >2 or more times (*p* = 0.003)	0.30
Slightly overweight	n	22	95	57					
	Hedonic hunger	2.78 ± 0.84	3.23 ± 0.78	3.50 ± 0.76	7.023	**0.001**	**0.078**	2 or more times > No consumption (*p* < 0.001)	0.92
								2 or more times > Less than 2 times (*p* = 0.016)	0.35
								Less than 2 times > No consumption (*p* = 0.030)	0.57
	HRQoL	32.86 ± 8.74	33.06 ± 7.27	31.74 ± 6.32	0.625	0.536	0.007		
Obesity	n	8	34	49					
	Hedonic hunger	3.21 ± 0.92	3.35 ± 0.98	3.73 ± 0.76	2.575	0.082	0.054		
	HRQoL	35.75 ± 7.21	34.69 ± 7.96	30.20 ± 6.62	4.713	**0.011**	**0.097**	No consumption >2 or more times (*p* = 0.008)	0.83
								Less than 2 times >2 or more times (*p* = 0.046)	0.62

HRQoL: Health-related quality of life. Significant *p* values are indicated in bold.

Age was positively associated with hedonic hunger (*B* = 0.059, *p* < 0.001), suggesting that older adolescents tend to experience higher levels of hedonic hunger. Gender had a significant effect, with males reporting lower hedonic hunger scores than females (*B* = −0.238, *p* < 0.001), highlighting gender differences in food reward mechanisms. BMI was not statistically significant (*B* = 0.004, *p* = 0.096), though a positive trend was observed, suggesting a potential but weak association between body mass index and hedonic hunger. Meal frequency showed mixed effects. Consuming three main meals per day was marginally associated with increased hedonic hunger (*B* = 0.234, *p* = 0.085), but the effect was not significant. Snacking more frequently (three times per day) was significantly associated with higher hedonic hunger (*B* = 0.336, *p* = 0.003). Sugary drink consumption had a dose-dependent effect. While consuming less than two glasses of sugary drinks per day was not significant (*B* = 0.028, *p* = 0.692), those who consumed two or more glasses had significantly higher hedonic hunger scores (*B* = 0.161, *p* = 0.032). Fast food consumption was strongly associated with hedonic hunger. Adolescents consuming fast food twice or more per week had significantly higher hedonic hunger scores (*B* = 0.558, *p* < 0.001), and even those who consumed it less than twice per week showed increased hedonic hunger (*B* = 0.278, *p* = 0.002). The multiple regression analysis investigated the predictors of health-related quality of life (HRQoL) scores. The model explained 17.3% of the variance in HRQoL (R^2^ = 0.173, Adjusted R^2^ = 0.162, *p* < 0.001). The Durbin-Watson statistic (1.790) suggests no serious autocorrelation issues. The regression model was statistically significant (*F*(12, 889) = 15.543, *p* < 0.001), indicating that the independent variables collectively contribute to explaining variations in HRQoL scores. Age was negatively associated with HRQoL (*B* = −0.909, *p* < 0.001), suggesting that as adolescents grow older, their perceived quality of life decreases. Gender had a significant effect, with males reporting higher HRQoL scores than females (*B* = 1.743, *p* = 0.001), highlighting gender differences in perceived well-being. Meal frequency showed mixed effects. Consuming two or three main meals per day was associated with higher HRQoL (*B* = 3.167, *p* = 0.007 and *B* = 4.502, *p* < 0.001, respectively). Similarly, more frequent snacking was positively associated with HRQoL, with significant effects for one snack per day (*B* = 2.700, *p* = 0.003), two snacks per day (*B* = 1.916, *p* = 0.034), and three snacks per day (*B* = 1.310, *p* = 0.175, though non-significant). Sugary drink consumption had a significant negative effect on HRQoL. Adolescents who consumed two or more glasses of sugary drinks per day reported lower HRQoL (B = −1.490, *p* = 0.019), whereas those consuming fewer sugary drinks did not show a significant decrease (*B* = −0.834, *p* = 0.162). Fast food consumption was also significantly associated with lower HRQoL. Adolescents consuming fast food twice or more per week reported significantly lower HRQoL scores (*B* = −2.844, *p* < 0.001), and those consuming it less than twice per week showed a negative but non-significant trend (*B* = −1.190, *p* = 0.111). BMI was marginally associated with lower HRQoL, but the effect was not statistically significant (*B* = −0.034, *p* = 0.062) (Table 5).

**Table 5:** Multiple regression analysis prediction hedonic hunger and HRQoL.

Predictor	B	SE	*t*	*p*	95% CI	
					Lower	Upper
Hedonic hunger						
(Intercept)	1.701	0.277	6.142	**< 0.001**	1.157	2.244
Age	0.059	0.013	4.545	**< 0.001**	0.034	0.085
Gender (Male)	−0.238	0.054	−4.378	**< 0.001**	−0.344	−0.131
BMI	0.004	0.002	1.665	0.096	−6.459 × 10^−4^	0.008
Number of Main Meals (2)	0.077	0.138	0.562	0.574	−0.193	0.347
Number of Main Meals (3)	0.234	0.136	1.725	0.085	−0.032	0.501
Number of Snacks (1)	−0.136	0.108	−1.257	0.209	−0.349	0.076
Number of Snacks (2)	0.028	0.107	0.264	0.792	−0.181	0.238
Number of Snacks (3)	0.336	0.114	2.940	**0.003**	0.112	0.560
Sugary Drinks Consumption (<2 glasses/day)	0.028	0.071	0.396	0.692	−0.111	0.166
Sugary Drinks Consumption (≥2 glasses/day)	0.161	0.075	2.143	**0.032**	0.014	0.309
Fast Food Consumption (<2 times/week)	0.278	0.088	3.143	**0.002**	0.104	0.451
Fast Food Consumption (≥2 times/week)	0.558	0.095	5.885	**< 0.001**	0.372	0.744
HRQoL						
(Intercept)	43.297	2.336	18.531	**< 0.001**	38.712	47.883
Age	−0.909	0.110	−8.269	**< 0.001**	−1.125	−0.694
Gender (Male)	1.743	0.459	3.800	**< 0.001**	0.843	2.643
BMI	−0.034	0.018	−1.866	0.062	−0.070	0.002
Number of Main Meals (2)	3.167	1.162	2.725	**0.007**	0.886	5.448
Number of Main Meals (3)	4.502	1.148	3.922	**< 0.001**	2.249	6.754
Number of Snacks (1)	2.700	0.915	2.950	**0.003**	0.904	4.497
Number of Snacks (2)	1.916	0.901	2.126	**0.034**	0.148	3.685
Number of Snacks (3)	1.310	0.964	1.359	0.175	−0.582	3.202
Sugary Drinks Consumption (<2 glasses/day)	−0.834	0.596	−1.400	0.162	−2.004	0.336
Sugary Drinks Consumption (≥2 glasses/day)	−1.490	0.636	−2.345	0.019	−2.738	−0.243
Fast Food Consumption (<2 times/week)	−1.190	0.746	−1.595	0.111	−2.654	0.274
Fast Food Consumption (≥2 times/week)	−2.844	0.801	−3.552	**< 0.001**	−4.415	−1.272

B = unstandardized coefficient, SE = standard error, t = t-statistic, *p* = significance level, 95% CI = confidence interval. Significant predictors (*p* < 0.05) are in bold.

## Discussion

To the best of our knowledge, there has been no research examining the effects of hedonic hunger on HRQoL in children and adolescents in the literature. We found that quality of life was negatively associated with the increase in hedonic hunger scores. We also examined hedonic hunger and HRQoL of adolescents aged 10–17 years according to the BMI z-score categories. Another key finding of the present study is that obese adolescents reported significantly higher levels of hedonic hunger than underweight and normal-weight adolescents. Additionally, we found an association between the consumption of sugary drinks and fast food with both quality of life and hedonic hunger in normal BMI levels. Given the cross-sectional nature of the study, causal inferences cannot be made, and it is important to note that our findings reflect associations rather than cause-and-effect relationships.

We also found that the female adolescents had more hedonic hunger than the male adolescents. Our finding regarding gender difference is similar to that of a study Stone et al. (18) conducted on 3,277 children. Nevertheless, the number of studies conducted on children is insufficient. Studies conducted on adults report that females may have higher levels of food craving (19). This craving may be linked to a psychological motivation to eat highly palatable foods, independent of the eating behavior. The samples of existing research in this area consist of few individuals or mostly females. Further research is needed to explore gender-based differences in the Children’s Power of Food Scale (18). The finding that females exhibit higher hedonic hunger scores than males may be influenced by several factors. Sociocultural influences could play a significant role, as women are often more exposed to societal pressures regarding body image and food-related behavior, which might lead to a heightened response to food cues. Hormonal variations, such as fluctuations in estrogen and progesterone levels, could also affect appetite regulation and increase susceptibility to hedonic hunger, especially during menstrual cycles. Additionally, females may be more prone to emotional eating tendencies, using food as a coping mechanism for stress or negative emotions. This may cause them to seek out highly palatable foods as a source of comfort, leading to higher hedonic hunger scores. Socialization around food and dieting practices may further reinforce these behaviors in females compared to males. These factors combined may help explain why females tend to report higher levels of hedonic hunger than their male counterparts (20).

Our study showed that the hedonic hunger status of the adolescents that were classified as obese according to their BMI-z scores was significantly higher compared to the underweight and normal-weight participants. A study examining the hedonic hunger and binge eating status of children and adolescents found a positive association between hedonic hunger scores and high BMI (>95th percentile) (3). Another study examining slightly overweight and obese adolescents stated that 16% of the participants had high hedonic hunger, and this was associated with increased calorie intake and higher BMI (21). Although the exact causes of hedonic hunger remain unclear, a study with a large sample of adolescents (*n* = 2,598) indicated that higher levels of negative urgency, general anxiety, and obsessive-compulsive symptoms increased the risk of hedonic hunger over a one-year follow-up (10). However, it is important to note that hedonic hunger may fluctuate during adolescence and differs from individual to individual (2). Given the observational nature of our study, causal relationships between hedonic hunger and obesity cannot be inferred, and further research is needed to explore these associations over time.

There was no significant difference in the KIDSCREEN scores according to the BMI categories of the participants (*p* > 0.05). A study conducted with European children and adolescents reported that the quality of life scores of slightly overweight children and adolescents were lower than normal-weight ones (22). Another study indicated that the quality of life scores of obese children and adolescents were lower than those of normal-weight ones. In addition, the study reported that the quality of life of children and adolescents whose parents were stressed decreased and their BMI increased (23). Considering this finding, families should also be evaluated while evaluating overweight/obese children and adolescents. Another study involving severely obese children and adolescents reported that lifestyle change treatment (nutrition, exercise and behavioral change) applied for 1 year increased the quality of life scores of the participants. In addition, a positive correlation was observed between more weight loss and an increase in the score during the treatment process (24). We believe the differences between our results and those in the literature may be influenced by factors such as the number of participants, ethnicity, and cultural differences. For instance, Turkish culture places a strong emphasis on family, community, and social acceptance, which could affect how adolescents perceive their weight and quality of life. Additionally, dietary habits, such as a preference for high-calorie, traditional foods and a relatively high intake of sugar and fat in the Turkish diet, could influence the relationship between BMI and HRQoL differently than in other populations (25, 26). These cultural and dietary factors may contribute to the variations in the observed associations between BMI and quality of life in our study compared to those reported in the literature.

Our study indicated that the consumption of fast food increased significantly as hedonic hunger scores increased in adolescents who were classified as normal weight or slightly overweight according to their BMI z-scores. Similarly, some studies have found a positive association between hedonic hunger and unhealthy food and beverage intake during adolescence (2, 27). There are limited studies on hedonic hunger in adolescents in Turkey (28). Kaur and Jensen (21) found that slightly overweight or adolescents with obesity (*n* = 100) with greater hedonic hunger had higher BMI. Considering these findings, it could be suggested that physiological and neurobiological developmental changes during adolescence may influence the relationship between hedonic hunger and fast food consumption (10).

Our study provided insight into the potential impact of body weight on the diet quality and food choices of adolescents. Adolescents with obesity who did not consume fast food had significantly higher HRQoL than those consuming it 2 or more times per week. This result were in line with previous studies that investigated the relationship between behavioral risk factors, diet quality, and HRQoL in adolescents (29–31). This finding highlights the potential negative impact of frequent fast-food consumption on health-related quality of life (HRQoL) in adolescents with obesity. Regular intake of fast food, often high in unhealthy fats, sugars, and additives, may contribute to poor physical health, increased metabolic risk, and lower overall well-being. In contrast, avoiding fast food may be associated with healthier dietary habits, better physical functioning, and improved psychological well-being. These results underscore the importance of promoting healthier eating patterns to enhance HRQoL in adolescents with obesity. Earlier research has shown that the consumption of high-energy fast food and sugary drinks is associated with unhappiness, poor sleep, perceived stress, and depression (32–35). These associations may reflect the harmful effects of fast food on both physical and mental health. Additionally, our findings are consistent with studies conducted in Western countries and some countries in Asia (30, 36), indicating that the relationship between food choices and HRQoL is relevant across different adolescent populations. However, due to the cross-sectional nature of our study, it is important to note that we cannot make causal inferences, and the observed associations should not be interpreted as evidence of causality.

Our study found that hedonic hunger more increased sugar consumption in adolescents with obesity than others (slightly overweight, normal BMI levels, underweight, malnutrition). This finding suggests that adolescents with obesity may have a stronger drive for palatable foods, particularly sugary items, due to heightened hedonic hunger. This increased susceptibility to food rewards could contribute to excessive sugar consumption, potentially exacerbating weight gain and related health risks in this population. In a prospective study (*n* = 3,268), hedonic hunger level of adolescents and sugary beverage consumption were found to be related, which is in line with our results. Another study conducted with adolescents found that hedonic hunger led adolescents to choose sugary and starchy foods. The study also reported that the HRQoL scores of the adolescents decreased with the increase in sugar consumption (2). It is known that there is a high level of evidence that the consumption of sugar and sugary drinks increases the risk of slight overweightness, obesity and comorbidities in children and adolescents.

Our study found no significant difference between the HRQoL scores of the adolescents, who were divided into five groups (malnutrition, underweight, normal-weight, slightly overweight, obesity) according to their BMI score, and it is not possible to comment on the HRQoL of the adolescents according to their weight. Other studies indicate that an increase in BMI is associated with a decrease in HRQoL (24, 37). It is understood that children and adolescents who are not of normal weight feel that they are unhealthy. In addition, our study found an association between increased sugar consumption and lower HRQoL scores in adolescents in the underweight and normal-weight groups, suggesting a potential negative impact on their quality of life. While this association could suggest that higher sugar intake is linked to poorer perceived health, Seum et al. (38) reported that sugar consumption and sugary drinks did not affect health outcomes, including HRQoL scores, which contradicts our findings. However, due to the observational nature of our study, it is important to acknowledge that these findings only show an association and not causality. Further research, including longitudinal studies, is needed to better understand the relationship between sugar consumption and HRQoL in adolescents. The lack of significant differences in HRQoL across BMI categories in our study could be explained by several factors. Psychosocial resilience, such as family support and coping mechanisms, may have helped adolescents mitigate the potential negative effects of BMI on quality of life. Additionally, the KIDSCREEN-10 tool may not capture the full range of factors influencing HRQoL, such as body image or social challenges specific to BMI groups. The sample in our study may have unique characteristics, such as socioeconomic status or cultural factors, that influenced HRQoL outcomes. These factors may differ from those in other studies that found a correlation between BMI and HRQoL. Thus, psychosocial factors, limitations in the measurement tool, and sample-specific influences could all contribute to the observed discrepancy.

Our findings suggest that gender, age, and dietary habits play a crucial role in hedonic hunger. The results indicate that females experience higher hedonic hunger than males, possibly due to differences in hormonal and psychological responses to food rewards. Age-related increases in hedonic hunger may reflect developmental changes in appetite regulation and reward sensitivity during adolescence. Notably, dietary behaviors such as frequent snacking, fast food intake, and high sugary drink consumption emerged as significant predictors of increased hedonic hunger. The strongest effects were observed for fast food intake, indicating a dose–response relationship in which more frequent consumption led to greater hedonic hunger. These results support the notion that dietary patterns characterized by processed and highly palatable foods reinforce reward-driven eating behaviors, potentially contributing to unhealthy eating habits and obesity risk. Although BMI was not a significant predictor, its positive trend suggests that higher BMI may still play a role in hedonic hunger, warranting further investigation. Future research should explore the underlying mechanisms linking hedonic hunger with weight status, particularly in adolescents, to better understand the implications for obesity prevention and dietary interventions.

Despite the significant results of our study, there are some limitations. The most important one is that the investigated parameters were evaluated only through self-report measures. While the measurements have good psychometric properties, they are prone to retrospective recall bias and measurement error. Future research may become more effective with the use of biological measurements (for example, neuroimaging) alongside clinical interviews. Another limitation is that The KIDSCREEN-10 tool may not fully capture weight-related issues, such as body image dissatisfaction or emotional eating, which are more pronounced in certain BMI categories. As a result, the lack of significant HRQoL differences could be due to the tool’s limited sensitivity to these specific challenges. In addition, considering the cross-sectional design of the study and the limitation of the sample to the Turks, the inability to generalize the findings to other populations is considered to be an important limitation. And also due to the lack of detailed data on mental health factors and socioeconomic status in the population, these potential confounders could not be directly accounted for in the analysis. While adjustments were made for available covariates, the absence of comprehensive information on these factors may limit the precision of the findings. Future studies should aim to incorporate more detailed information on these variables to better control for potential confounding effects.

## Conclusion

Our results indicate that hedonic hunger is associated with higher BMI in adolescents. They also show that hedonic hunger and HRQoL are important factors associated with unhealthy food and beverage intake. Hedonic hunger may have crucial implications in preventing obesity and promoting health in adolescence. Evaluation of hedonic hunger during the school period may be of particular significance in identifying young individuals who are at risk of increased consumption of unhealthy foods and beverages. In addition, strategies to reduce hedonic hunger in adolescents or to cope with the urge to consume highly palatable foods can reduce their consumption of unhealthy foods and beverages. Further studies are required to elucidate the risk factors for hedonic hunger and HRQoL in childhood and adolescence. To address hedonic hunger and its impact on adolescent obesity, public health interventions could include mindful eating programs that help adolescents become more aware of their hunger cues and eating habits, promoting a healthier relationship with food. These programs could teach techniques such as paying attention to food textures and flavors, eating without distractions, and recognizing emotional triggers for overeating. Additionally, nutritional education focused on food reward mechanisms can educate adolescents about how highly palatable foods activate the brain’s reward system, leading to overeating. This could empower them to make more informed choices and reduce reliance on food for pleasure. Lastly, behavioral strategies aimed at promoting healthier eating habits might include goal-setting, self-monitoring, and positive reinforcement techniques to encourage consistent, nutritious food choices while reducing impulsive or emotional eating. Together, these strategies could help mitigate the influence of hedonic hunger on adolescent obesity.

## Data Availability

The original contributions presented in the study are included in the article/Supplementary material, further inquiries can be directed to the corresponding author.
