# Supplementary figures and images for: Are hedonic hunger and health-related quality of life associated with obesity in adolescents?
Source: Front Nutr. 2025 Mar 26;12:1557765. doi: 10.3389/fnut.2025.1557765 (PMC11978664; doi:10.3389/fnut.2025.1557765)

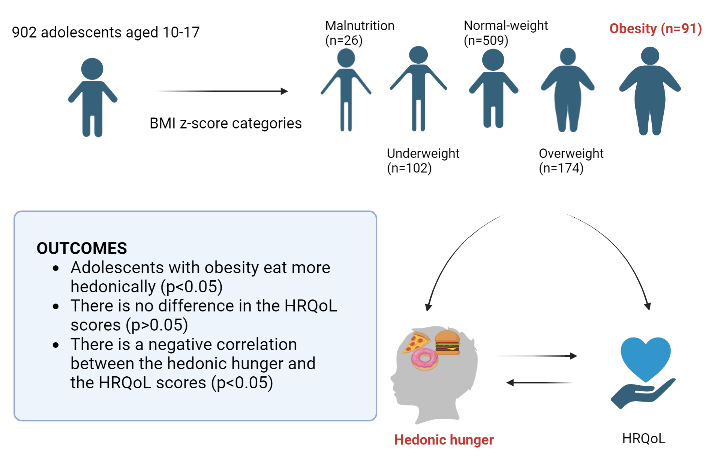

Supplement: Supplementary file 1 [file Image_1.png]
